# Effects of Probiotic Supplementation on Mental Health and the Risk of Depression in Women with Polycystic Ovary Syndrome: A Systematic Review of Randomized Controlled Trials

**DOI:** 10.3390/nu18020307

**Published:** 2026-01-19

**Authors:** Karolina Łagowska, Dagmara Ptaszyńska

**Affiliations:** Department of Human Nutrition and Dietetics, Poznań University of Life Sciences, Wojska Polskiego 31, 60-624 Poznań, Poland; karolina.lagowska@up.poznan.pl

**Keywords:** polycystic ovary syndrome, probiotic supplementation, depression, anxiety, sleep quality

## Abstract

**Background:** Polycystic ovary syndrome (PCOS) is commonly associated with psychological disorders, including depression and anxiety. Women with PCOS also tend to experience poorer sleep quality and greater daytime sleepiness than healthy individuals. To the best of our knowledge, no systematic reviews have investigated the impact of probiotic supplementation on mental health and sleep patterns in women with PCOS. Emerging evidence indicates that probiotic therapy may be a promising adjunct for enhancing mental well-being and sleep quality within this population. **Objectives**: This systematic review aimed to evaluate the effects of probiotic supplementation on depression, anxiety, and sleep quality in adult women with PCOS. **Methods**: PubMed, Cochrane, and Scopus were searched for randomized controlled trials (RCTs) involving women aged 18–45 years old, with diagnosed PCOS, who received probiotic/synbiotic supplements and enriched foods compared with placebo. Studies had to assess mental health, depressive symptoms, or sleep disorders using validated questionnaires. Five publications met the Population, Intervention, Comparison, and Outcome inclusion criteria (PICO) and were included in the final analysis. **Results**: Probiotic supplementation was associated with significant improvements in sleep quality (assessed by Pittsburgh Sleep Quality Index, PSQI), depressive symptoms (assessed by Depression, Anxiety and Stress Scale, DASS 21) and some domains of quality of life (measured by Polycystic Ovary Syndrome Health Related Quality of Life Questionnaire, PCOSQ-26). **Conclusions**: Although probiotic supplementation may benefit mental health and sleep parameters in women with PCOS, this evidence is limited due to the small number of studies, modest sample sizes, and methodological variability. Further research with larger, more rigorous studies is needed to confirm these findings.

## 1. Introduction

Polycystic ovary syndrome (PCOS) represents a common endocrine-metabolic disorder, seen in approximately 5–10% of women of reproductive age [1]. PCOS is associated with a wide range of complications, including hormonal and metabolic disorders such as visceral obesity, insulin resistance and hyperinsulinemia, dyslipidemia, cardiovascular diseases, endometrial cancer, infertility, and adverse pregnancy outcomes, and is also linked to low-grade inflammation, which enhances ovarian androgen production, resulting in increased oxidative stress [2]. PCOS is a major public health burden. Beyond the physical health complications, women with PCOS also face significant psychological challenges, including heightened risk for depression and anxiety. Women characterized by hyperandrogenic features such as hirsutism—present in up to 60% of women with PCOS—tend to have a greater risk of elevated tension, enhanced social anxiety, and, in some cases, suicidal thoughts [3]. It has also been suggested that women with PCOS have poorer sleep quality, greater daytime sleepiness, and lower overall sleep efficiency when compared to healthy participants [4,5]. The risk of depression is much higher in women with PCOS and advanced insulin resistance. In addition, women with PCOS struggling with infertility report a particularly low quality of life. Moreover, depression in women with PCOS is often linked to reduced cognitive function, with studies indicating impairments in memory and attention, particularly in the domain of episodic memory. Low verbal-episodic-memory performance is considered a premorbid marker of depression. Global standards describing the treatment of women with PCOS emphasize the need for screening for depression at the time of diagnosis of PCOS, which also reflects the growing concerns of doctors related to this aspect of the disease.

There is a need to develop new therapeutic approaches to mitigate the consequences of these conditions. In recent years, the efficacy of probiotic supplementation for PCOS treatment has been demonstrated in numerous studies [6,7,8,9,10,11]. Probiotics may also improve several cognitive domains, including verbal episodic memory, in healthy participants and patients with Alzheimer’s disease or other cognitive impairments [12,13]. This may have important implications for the population of women with PCOS because this syndrome is associated with multiple factors affecting cognition, including metabolic syndrome, diabetes, and depression, potentially mediated by the dysregulation of the brain–ovary axis and the AMH-related modulation of steroid hormone synthesis. Neuroimaging studies in women with PCOS further demonstrate reduced global and regional brain volumes, altered white matter microstructure, and disrupted connectivity, particularly in frontal and parietal regions [14,15,16]. Also in a study by Zandifar et al., the potential positive effects of probiotic supplementation on cognitive functions were confirmed [17]. Probiotics are also unlikely to produce the cognitive side effects or the addictive potential associated with several currently used pharmacological treatments for these disorders [18]. This systematic review of randomized controlled trials (RCTs) aims to assess the potential benefits of probiotic supplementation on mental health outcomes in women with PCOS.

## 2. Materials and Methods

This section was organized as follows: First, the inclusion criteria for studies were defined based on the Population, Intervention, Comparison, and Outcome inclusion criteria (PICO) framework. Subsequently, scientific databases were searched using predefined keywords. The final step involved data extraction followed by data analysis. Mental health, depressive symptoms, and sleep disorders were predefined as primary outcomes, and general health was considered a secondary outcome.

### 2.1. Search Strategy and Inclusion Criteria

Two authors (KŁ and DP) independently conducted all the literature searches and quality assessments in duplicate, following a standardized methodology. Data extraction was conducted by one author, with problems resolved by a second. This systematic review protocol has been registered in the International Prospective Register of Systematic Reviews (PROSPERO) under the number CRD420251148133. This systematic review fully complies with the Preferred Reporting Items for Systematic Reviews (PRISMA 2020) guidelines, and the PRISMA checklist is provided in Supplementary Materials [19] (Supplementary File S1). The PICO (Population, Intervention, Control, and Outcome) strategy was used to construct the research question as summarized in Table 1.

### 2.2. Literature Sources, Search Strategy, and Selection Criteria

**Figure 1:**
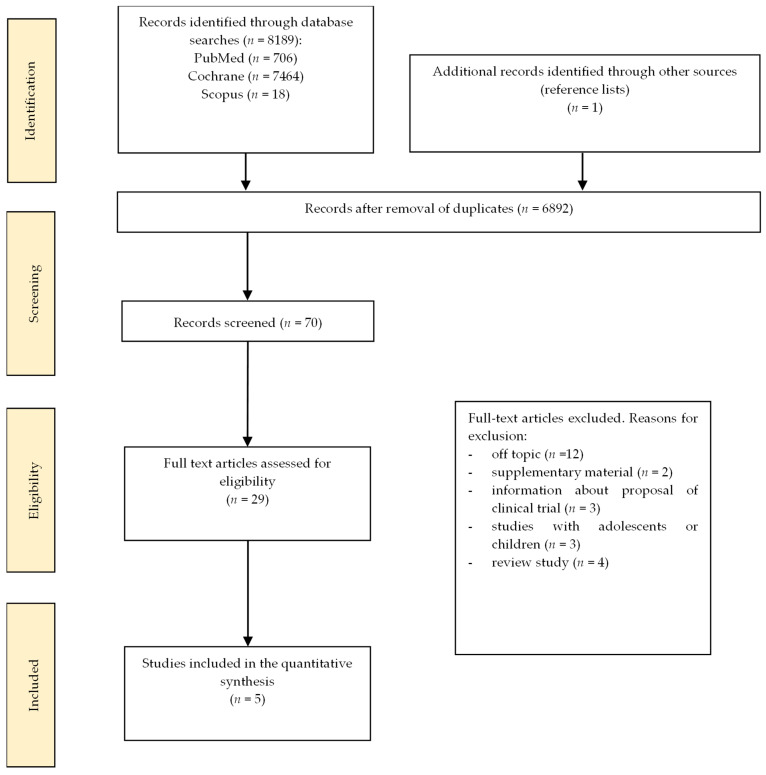
Flow diagram of the study inclusion.

Three databases—PubMed, Scopus, and Cochrane—were searched up to September 2025 using a predefined search strategy combining terms related to probiotics (“probiotic” OR “synbiotic”), polycystic ovary syndrome (“polycystic ovary syndrome” OR “PCOS”), and mental health outcomes (“depression” OR “mental health” OR “depressive symptoms” OR “sleep disorders”), connected with Boolean operators AND/OR. The full searching phrase was as follows: (“probiotics” or probiotic* OR symbiotic*) AND (“polycystic ovary syndrome” OR PCOS) AND (depression OR depressive symptoms OR mental health OR sleep disorders). The inclusion criteria were as follows: RCTs comparing the effects of probiotic/synbiotic supplementation in PCOS with placebo; any date of publication; studies involving women aged 18–45 with a confirmed diagnosis of PCOS; articles published in English in peer-reviewed journals; and sufficient extractable data. The age range of 18–45 years was selected because PCOS is diagnosed exclusively in women of reproductive age, for whom diagnostic criteria are most reliable. Exclusion of adolescents and perimenopausal women minimizes hormonal variability that could confound mood and sleep outcomes. Additionally, this age range is commonly used in clinical studies regarding PCOS, ensuring comparability with the existing literature. Detailed reasons for exclusion are presented in Figure 1. Reviews, experiments with animal models, observational studies, master’s and doctoral theses, commentaries, conference materials, and letters were excluded from the analysis. Studies without a control/placebo group were excluded.

### 2.3. Data Extraction and Analysis

Key study characteristics, including first author, publication year, country, supplementation and placebo details, duration of the probiotic/symbiotic intervention, and sample size, were assessed independently by one reviewer (KL) with any issues resolved by consensus by a second reviewer (DP). Study eligibility was independently assessed by two authors (KL, DP), and any discrepancies were resolved through discussion. When outcome data were missing, two attempts were made by KL to reach out to the study authors for the relevant details.

The participant characteristics were mean age or age range and body mass index (BMI; kg/m^2^), and the main outcomes were mental health, depressive symptoms, and sleep disorders. If more than one follow-up time point was reported, the data from the last follow-up were used. Authors independently assessed the risk of bias in each study using the latest version of the Cochrane Collaboration Risk of Bias tool for RCTs with parallel groups (RoB 2) [20]. Studies were evaluated across the following five domains for parallel-group trials: bias arising from the randomization process; bias due to deviations from the intended interventions; bias due to missing outcome data; bias in the measurement of the outcome; and bias in selecting the reported results. The tool includes algorithms that map responses to signaling questions to a proposed risk-of-bias judgment for each domain, across three levels: low or high risk of bias and some concerns.

## 3. Results

### 3.1. Search Results

A flowchart of the study extraction process is presented in Figure 1. Initially, 8189 articles were identified, of which 29 were selected for full-text review. Finally, five publications met the inclusion criteria and were included in the final analysis.

### 3.2. Population and Study Characteristics

The risk of bias assessment revealed variability in the methodological quality of the included studies. Two studies were judged to have a high overall risk of bias [9,21], primarily due to concerns related to the randomization process and the measurement of outcomes, as well as selective reporting of results. The remaining studies were assessed as having some concerns [6,22,23], most commonly associated with incomplete reporting of randomization procedures, missing outcome data, or potential deviations from intended interventions. Overall, domains related to deviations from intended interventions were consistently -marked as low risk, whereas issues concerning outcome measurement and reporting were the most frequent sources of bias. Disagreements between authors were resolved by discussion. Overall risk of bias judgment was used to evaluate each study. A detailed domain-level assessment for each study is presented in Supplementary Table S1. Due to the limited number of studies included in this systematic review, all studies were treated equally regardless of their quality assessment results. This assessment suggests that conclusions based on the cited studies should be approached with caution.

Table 2 summarizes the studies and their participants. The articles were published between 2018 [9] and 2025 [22]. All studies were conducted in Asia, with four in Iran [6,9,21,22] and one in India [23]. This may limit the generalizability to other populations. The number of participants ranged from 28 [21] to 48 [23] in the supplementation groups and from 24 [21] to 49 [23] in the placebo groups. The mean age (years) of participants ranged from 23.6 ± 3.9 [23] to 33.5 ± 5.5 [22] in the supplemented groups and from 24.4 ± 4.8 [23] to 33.4 ± 5.5 [22] in the placebo groups. Their BMI (kg/m^2^) ranged from 24.6 ± 3.3 [9] to 28.6 ± 4.8 [22] in the supplemented groups and from 24.0 ± 3.0 [9] to 28.7 ± 4.8 [22] in the placebo groups. All studies used a parallel design, with one comparing yogurt fortified with probiotic bacteria + 50 IU vitamin E + 1000 IU vitamin D against low-fat yogurt without probiotic bacteria [22]. In the remaining four studies, patients were administered a capsule or sachets and compared with the placebo group [6,9,21,23]. Supplementation protocols differed substantially in the examined, resulting in high heterogeneity.

The duration of the intervention varied across studies, from 8 weeks [22] to 6 months [23]. The participants’ mental health was evaluated using validated questionnaires: Depression, Anxiety, and Stress Scale-21 items or Depression, Anxiety, and Stress Scale (DASS-21 or DASS) [6,9,22]; Polycystic Ovary Syndrome Health-related Quality of Life Questionnaire or Modified Polycystic Ovary Syndrome Health-related Quality of Life Questionnaire (PCOSQ-26, mPCOSQ) [21,23]; 12-item Short-form Health Survey (SF-12) [21]; Perceived Stress Scale (PSS-10) [21]; Beck Depression Inventory (BDI) [6,9]; and General Health Questionnaire-28 (GHQ-28) [6,9]. Sleep quality was evaluated using the Pittsburgh Sleep Quality Index (PSQI) [6,22].

### 3.3. Quality of Sleep (PSQI)

Two studies evaluated sleep profile using the PSQI [6,22]. Askarpour et al. [22] observed a significant improvement in sleep quality in the SG after 8 weeks, with no change in the PG. Ostadmohammadi et al. [6] did not show the results in groups. In both studies, the improvement was not statistically significant between PG and SG (Table 3).

### 3.4. Depression, Anxiety, and Stress Scale 21 Items, Depression, Anxiety, and Stress Scale (DASS-21, DASS)

Three studies used the DASS-21 or DASS questionnaire for evaluating mental health among women with PCOS [6,9,22]. In the study of Askarpour et al. [22], the outcomes were reported in the subscales of anxiety, depression, and psychological scores. In the remaining studies, authors presented composite scores [6,9]. Askarpour et al. [22] reported a significant difference in depressive symptoms in women with PCOS after 8 weeks of probiotic intervention. Furthermore, depressive symptoms were lower in the supplemented group (SG) compared to the placebo group (PG) at the end of the intervention. There were no significant differences after probiotic supplementation in the anxiety and psychological scores [22]. Jamilian et al. [9] and Ostadmohammadi et al. [6] reported all symptoms (depressive symptoms, anxiety, psychological score) combined, and both studies showed significant improvement after probiotic supplementation compared with placebo. All of these studies suggest an improvement in the risk of depression after probiotic supplementation (Table 3).

### 3.5. Polycystic Ovary Syndrome Health-Related Quality of Life Questionnaire or Modified Polycystic Ovary Syndrome Health-Related Quality of Life Questionnaire (PCOSQ-26, mPCOSQ)

Two studies used the PCOSQ-26/PCOSQm to evaluate health and quality of life among women with PCOS [21,23]. Hariri et al. [21] reported that synbiotic supplementation improved scores in the emotional, body hair/hirsutism, weight, and infertility domains compared to PG. Kaur et al. [23] observed significant improvements in all domains (emotions, body hair/hirsutism, weight, infertility, and menstrual) in both group (SG and PG) at the end of the trial. Differences between the SG and PG (at the end of the intervention) were observed only in menstrual problems domains [23]. In study of Hariri et al. [21] showed significant differences in emotions and infertility domain in SG at the end of the intervention. No differences between the SG and PG (at the end of the intervention) were observed in case of body hair/hirsutism, weight and infertility domain.

Both study protocols vary substantially. In the study of Kaur et al. [23], the intervention included not only symbiotic supplementation but also dietary modifications and physical activity, whereas Hariri et al. [21] investigated the effects of supplementation alone. Additionally, Hariri et al. [21] administered a constant symbiotic dose throughout the study, while Kaur et al. [23] employed a dose that varied over time. Both studies suggest that synbiotic supplementation may have a potential positive impact in terms of the areas measured by the Polycystic Ovary Syndrome Health-Related Quality of Life Questionnaire. The consistency of these two studies contrasts with the more variable findings reported for other mental health measures, highlighting a potential domain-specific effect of the intervention.

### 3.6. Beck Depression Inventory (BDI)

Two studies evaluated the risk of depression using the BDI [6,9]. In both studies, probiotic supplementation resulted in a significant improvement in BDI compared with the placebo, where the risk of depression in the SG was lower than in PG at the end of the intervention (Table 3). 

### 3.7. General Health Questionnaire (GHQ)

Two studies assessed mental health using the GHQ [6,9]. In both studies, probiotic supplementation resulted in a significant improvement in GHQ (psychiatric conditions) compared with the placebo at the end of the trial (Table 3).

### 3.8. 12-Item Short Form Health Survey (SF-12)

One study used the SF-12 to measure health-related quality of life, but reported no significant differences at the end of the 12-week intervention between groups [21]. This may result from the small sample sizes of the study groups or the potentially limited sensitivity of the SF-12 questionnaire to detect changes (Table 3).

### 3.9. Perceived Stress Scale (PSS-10)

One study used the PSS-10 to measure the degree to which situations in one’s life are appraised as stressful, but observed no significant improvement at the end of the 12-week intervention [21] (Table 3).

### 3.10. Comments on Used Questionnaires and Insight on Their Characteristics

Although some studies found no significant effects of probiotics on anxiety or psychological stress, these null findings contrast with observed improvements in depressive symptoms, suggesting potential domain-specific benefits. Moreover, some overlap between the constructs measured by BDI and DASS-21 should be noted when interpreting these consistent findings.

Also, differences between DASS-21 and PSS-10 findings may reflect their distinct approaches. The DASS-21 questionnaire provides separate scores for depression, anxiety, and stress based on symptom frequency over the past week, whereas PSS-10 assesses overall perceived stress and sense of control over the past four weeks.

## 4. Discussion

### 4.1. The Significance of This Systematic Review

This systematic review provides an overview of five RCTs published between 2018 and 2025, examining the possible effects of probiotic supplementation on mental health and sleep patterns in individuals with PCOS, and represents the first review to specifically integrate RCT data on these outcomes, thereby highlighting its novel contribution.

### 4.2. Gut Microbiota and Depression Prevalence—Mechanism of Action

Growing attention has been directed toward the gut microbiota–brain axis as a potential therapeutic approach for depression, as evidence consistently indicates differences in microbiota composition between patients with depressive symptoms and healthy individuals. Recent studies have linked these gut microbiota alterations to a reduction in anti-inflammatory, butyrate-producing bacteria and an increase in pro-inflammatory bacteria in depressed patients [24]. Additionally, higher levels of butyrate-producing genera, such as *Faecalibacterium* and *Coprococcus*, have been correlated with better quality-of-life indicators [25]. Schneider et al. [26] reported that short-term, high-dose probiotic supplementation in depressed individuals led to significant reductions in depression scores and helped to stabilize microbial diversity and richness. Our systematic review was restricted to women with PCOS, as numerous authors have reported that depression occurs three to four times more frequently in this population compared to healthy controls [27]. The possible explanation is that women with PCOS more frequently present inflammation, insulin resistance, and hyperandrogenism compared to healthy women, which are factors that contribute to a higher risk of depression. Heightened risk of depression in this group may also result from dysregulations in endocrine and immune systems and gut microbiota disturbances [28]. Furthermore, no literature review to date has summarized the influence of probiotic supplementation on mental health within this population.

### 4.3. Sleep Disturbances as Symptoms of Depression

One of the main symptoms of depression is a sleep disorder. Many studies indicated that sleep disturbances, such as difficulty sleeping, excessive daytime sleepiness, disturbed sleep, insomnia, or obstructive sleep apnea (OSA), are more common among women with PCOS [29]. Jafar et al. [30], in the last meta-analysis, showed that 46.0% of women with PCOS had OSA and 56.0% had other sleep disruptions. It could be due to alterations in the neurometabolic profile typical of this disorder, as this system plays a crucial role in controlling the sleep–wake cycle by regulating melatonin and cortisol secretion [31]. Other studies suggested that sleep disturbances may also be associated with decreased SHBG levels [30,32].

### 4.4. Probiotic Supplementation as a Potential Therapy for Sleep Disorders

Moreover, sleep problems have been found to affect reproductive function [33], psychological health, and lifestyle habits [34]. In this systematic review, only two studies addressed sleep quality assessment. Askarpour et al. [22] reported a significant improvement in sleep quality after 8 weeks of consuming yogurt fortified with 106 colony-forming units/g of probiotics (*Bifidobacterium animalis* Bb-12 and *Lactobacillus acidophilus* La-5) in combination with 50 IU of vitamin E and 1000 IU of vitamin D. However, there were no differences between the SG and PG (at the end of the intervention); therefore, the results should be considered with caution. In other populations, many authors have reported that probiotic supplementation has been effective in treating sleep disorders. According to Ho et al. [35], 30-day supplementation of *Lactobacillus plantarum* PS128 enhanced sleep quality in insomniac individuals aged 20–40 years.

### 4.5. The Mental Health Outcomes

Depressive symptoms have also been linked with anxiety and a negative perception of the future [36]. Other authors indicated that it could be related to feelings of female attractiveness and fertility [37,38]. The symptoms of PCOS, including acne, androgenic alopecia, obesity, hirsutism, and other symptoms, lead to a reduction in feminine traits and cause patients to view themselves as less attractive, thereby adversely impacting their mental health through reduced self-esteem and body satisfaction [37,38]. Studies included in our systematic review evaluated depressive symptoms using, among others, the Polycystic Ovary Syndrome Health-related Quality of Life Questionnaire, where depressive symptoms include questions from five domains: emotions, body hair/hirsutism, body weight, infertility, and menstrual dysfunctions. These domains align with observations from other authors, who have highlighted that abdominal obesity, poor mental well-being, hirsutism, and menstrual function disorders are associated with increased anxiety in women with PCOS [37,38]. It should be stressed that the assessment of the effects of probiotic supplementation does not clearly indicate improvement in these domains. Although notable progress was sometimes seen in the SG, comparison of results between the SG and PG at the end of supplementation often revealed no significant differences. Also in the study of Kaur et al. [23], both the SG and PG improved in several domains, which may suggest potential effects of co-interventions or placebo response. This conclusion may be extended to the other studies discussed.

### 4.6. Strain-Specific Considerations

Authors also measured changes in depressive symptom severity and general health in women with PCOS using the BDI and GHQ after supplementation with *Lactobacillus acidophilus*, *Bifidobacterium bifidum*, *Lactobacillus reuteri*, and *Lactobacillus fermentum* [6,9]. In both studies, significant improvement was observed after the intervention. The BDI and GHQ may be more sensitive to changes than the PCOSQ-26 and PSS-10 because they target clinically relevant symptoms of depression and psychological distress, allowing for a finer discrimination of symptom severity. Conversely, the PCOSQ-26 and PSS-10 emphasize subjective perceptions of life quality and stress, which may be influenced by multiple factors and thus show lower responsiveness to change. Comparable results were also observed by Sanchez et al. [39] who reported a decrease in BDI score in women with obesity (but without PCOS) supplemented with *Lactobacillus rhamnosus* CGMCC1.3724 (*p* = 0.002). Raygan et al. [40] reported that the BDI (*p* = 0.01) and Beck Anxiety Inventory (BAI) (*p* = 0.009) scores were significantly reduced by supplementing a combination of probiotic and vitamin D (50,000 IU). However, as the study included both female and male participants, the independent effect of probiotic supplementation remains uncertain. Ahmad et al. [41] administered *Lactobacillus rhamnosus* GG (10B CFU) in combination with *Bifidobacterium longum* (5B CFU) or placebo to obese patients with moderate depression, insomnia, and anxiety. It should also be emphasized that in numerous studies investigating probiotic administration in the context of depressive symptoms and sleep disturbances across different populations, *Lactobacillus* and *Bifidobacterium*, alone or in combination with prebiotics or other probiotics, were the most frequently employed bacterial strains [42,43]. *Lactobacillus rhamnosus* is among the earliest and most thoroughly investigated probiotic species within the *Lactobacillus* genus, demonstrating considerable potential for the prevention and management of depression, and is among the most frequently associated with favorable outcomes [42]. The main probiotic strains in all studies incorporated into this systematic review were *Lactobacillus* and *Bifidobacterium*. However, considering that the majority of available study results in women with PCOS do not clearly demonstrate that probiotic supplementation is an effective strategy for reducing stress, anxiety, and sleep disorders in this population, further research is necessary. However, it is difficult to attribute positive effects of supplementation solely to probiotics due to high heterogeneity in study protocols, which include not only probiotics but also co-supplementation with vitamins and lifestyle interventions. These studies should focus not only on the effects of probiotic supplementation as part of the treatment for mental health disorders, but also on identifying which strains may be most effective in this context. They should also exclude other factors that may influence the outcomes, including simultaneous vitamin supplementation, dietary changes, and changes in physical activity during intervention. The existing evidence on mood and sleep disorders in women with PCOS remains limited. As this research area continues to evolve, conclusions and cross-population comparisons should be made with caution to avoid unwarranted generalization.

## 5. Conclusions

This study has several strengths. To the best of our knowledge, this is the first systematic review to investigate the impact of probiotic supplementation on mental health outcomes and sleep patterns in women with PCOS. Focusing on this topic is clinically relevant given the high burden of psychological comorbidities among women with PCOS. Nevertheless, this review is subject to several limitations, which, taken together, preclude definitive conclusions and do not allow for clear clinical recommendations regarding the use of probiotics for mental health or sleep outcomes in women with PCOS. First, the number of eligible studies was limited, and all were weighted equally despite different outcomes in the risk of bias assessment. In addition, the majority of trials involved small sample sizes with short intervention durations. Second, differences in dosage, probiotic strains, formulation, and mental health and sleep assessment methods hinder clear interpretation of the intervention’s clinical effects. Although studies on obese patients and women without PCOS have shown promising effects of probiotic supplementation on mental health and sleep quality, standardized supplementation protocols and robust methodological approaches are still needed to effectively assess these outcomes in women with PCOS. Further research employing larger cohorts, longer follow-up, standardized outcome measures, strain-specific effects, and optimal dosage is needed.

## Figures and Tables

**Table 1:** PICO criteria for inclusion of studies.

PICO Criteria	Definition of Criteria for Studies
Participants	women in reproductive age (aged 18–45) with PCOS diagnosis based on the Rotterdam criteria
Intervention/exposure	probiotic/synbiotic supplementation and/or foods enriched with probiotic/synbiotic
Comparator	placebo
Outcomes	questionnaires assessing mental health (PCOSQ-26, PSS-10), depressive symptoms (DASS-21, BDI), quality of sleep (PSQI), general health and quality of life (GHQ, SF-12)

**Table 2:** Details of studies of the effects of probiotic supplementation on mental health and quality of sleep in women with PCOS.

Study	Country	Type of Intervention	Side Effect	Time of Intervention	Study Group	Age (Years)	BMI (kg/m^2^)	Questionnaires
Askarpour et al., 2025 [22]	Iran	* SG: multispecies probiotic yogurt fortified with vitamin E + D PG: low-fat yogurt	No	8 weeks	SG = 40 PG = 41	SG = 33.5 (5.5) PG = 33.4 (5.5)	SG = 28.6 (4.8) PG = 28.7 (4.8)	Pittsburgh Sleep Quality Index (PSQI) Depression, Anxiety, and Stress Scale-21 Items (DASS-21)
Hariri et al., 2024 [21]	Iran	** SG: multistrain probiotic fructooligosaccharide + natural orange flavoring ** PG: sachets filled with starch and natural orange flavoring	No	12 weeks	SG = 28 PG = 24	SG = 28.4 (6.1) PG = 32.8 (15.9)	SG = 25.2 (4.1) PG = 24.9 (4.3)	Polycystic Ovary Syndrome Health-related Quality of Life Questionnaire (PCOSQ-26), 12-item Short Form Health Survey (SF-12) Perceived Stress Scale (PSS-10)
Jamilian et al., 2018 [9]	Iran	*** SG: multistrain probiotic + selenium PG: placebo capsules	NR	12 weeks	SG = 30 PG = 30	SG = 26.0 (5.3) PG = 25.6 (3.8)	SG = 24.6 (3.3) PG = 24.0 (3.0)	Beck Depression Inventory (BDI), General Health Questionnaire-28 (GHQ-28) Depression, Anxiety, and Stress Scale (DASS)
Kaur et al., 2022 [23]	India	**** SG: multistrain probiotic + fructooligosaccharide + conventional healthy diet plans + two sets of exercise plans **** PG: placebo capsules + conventional healthy diet plans + two sets of exercise plans	No	6 months	SG = 48 PG = 49	SG = 23.6 (3.9) PG = 24.4 (4.8)	SG = 26.4 (5.5) PG = 27.6 (4.7)	Polycystic Ovary Syndrome Health-Related Quality of Life Modified Questionnaire (mPCOSQ)
Ostadmohammadi et al., 2019 [6]	Iran	***** SG: multistrain probiotic + vitamin D every 2 weeks PG: placebo capsules	No	12 weeks	SG = 30 IG = 30	SG: 24.4 (4.7) PG: 25.4 (5.1)	SG = 24.3 (4.2) PG = 25.1 (4.9)	Beck Depression Inventory (BDI), Depression, Anxiety, and Stress Scale (DASS), General Health Questionnaire (GHQ-28), Pittsburgh Sleep Quality Index (PSQI)

SG—study group, PG—placebo group, NR—not reported, CFU—colony-forming unit. * SG yogurt fortified with 10^6^ CFU/g of *Bifidobacterium animalis* Bb-12 and *Lactobacillus acidophilus* La-5 + 50 IU of vitamin E + 1000 IU of vitamin D. ** SG 10^11^ spores of *Bacillus coagulans* (*GBI-30*), 10^10^ CFU of *Lactobacillus rhamnosus*, 10^10^ CFU of *Lactobacillus helveticus*, + 500 mg fructooligosaccharide + 0.7% natural orange flavoring. PG sachets filled with starch and 0,7% natural orange flavoring. *** SG 8 × 10^9^ CFU/day of *Lactobacillus acidophilus*, *Lactobacillus reuteri*, *Lactobacillus fermentum*, and *Bifidobacterium bifidum* (2 × 10^9^ CFU/g each) + 200 μg/day selenium. **** SG 10^9^ CFU/day of *Lactobacillus acidophilus* UBLA-34, *L. rhamnosus* UBLR-58, *L. reuteri* UBLRu-87, *L. plantarum* UBLP-40, *L. casei* UBLC-42, *L. fermentum* UBLF-31, *Bifidobacterium bifidum* UBBB-55, and 100 mg of fructooligosaccharide + conventional healthy diet plans (carbohydrate (55–60%), fat (20%), and proteins (20–25%)) + two sets of exercise plans. PG placebo capsules with maltodextrin + conventional healthy diet plans (carbohydrate (55–60%), fat (20%), and proteins (20–25%)) + two sets of exercise plans. ***** SG 8 × 10^9^ CFU/day of *Lactobacillus acidophilus*, *Bifidobacterium bifidum*, *Lactobacillus reuteri*, and *Lactobacillus fermentum* (2 × 10^9^ CFU/g each) *+* 50,000 IU vitamin D every 2 weeks.

**Table 3:** Effect of probiotic supplementation on mental health and sleep profile among women with PCOS.

Study	Differences Between Baseline and the End of the Intervention in SG and PG	Differences Between SG and PG (at the End of the Intervention)
Quality of Sleep (PSQI)		
Askarpour et al., 2025 [22]	SG: ↓ PG: ↔	↔ ^1^
Ostadmohammadi et al., 2019 [6]	NR	↔ ^1^
Depression, Anxiety, and Stress Scale-21 Items (DASS-21)		
Askarpour et al., 2025 [22]	Depressive symptoms	Depressive symptoms
	SG: ↓ PG: ↔	↓ ^1^
	Anxiety and Psychological score	Anxiety and Psychological score
	SG: ↔ PG: ↔	↔ ^1^
	All symptoms counted together	
Jamilian et al., 2018 [9]	NR	↓ ^1^
Ostadmohammadi et al., 2019 [6]	NR	↓ ^1^
Polycystic Ovary Syndrome Health-related Quality of Life Questionnaire (PCOSQ-26)		
	Emotions domain (both studies)	Emotions domain (both studies)
Hariri et al., 2024 [21]	SG: ↑ PG: ↑	↔ ^1^
Kaur et al., 2022 [23]		
	Body hair/hirsutism domain	Body hair/hirsutism domain
Hariri et al., 2024 [21]	SG: ↔ PG: ↑	↔ ^1^
Kaur et al., 2022 [23]	SG: ↑ PG: ↑	↔ ^1^
	Weight domain	Weight domain
Hariri et al., 2024 [21]	SG: ↔ PG: ↑	↔ ^1^
Kaur et al., 2022 [23]	SG: ↑ PG: ↑	↔ ^1^
	Infertility domain	Infertility domain
Hariri et al., 2024 [21]	SG: ↑ PG: ↔	↔ ^1^
Kaur et al., 2022 [23]	SG: ↑ PG: ↑	↔ ^1^
	Menstrual problems	Menstrual problems
Hariri et al., 2024 [21]	SG: ↔ PG: ↔	↔ ^1^
Kaur et al., 2022 [23]	SG: ↑ PG: ↑	↑ ^1^
Beck Depression Inventory (BDI)		
Jamilian et al., 2018 [9]	NR	↓ ^1^
Ostadmohammadi et al., 2019 [6]	NR	↓ ^1^
General Health Questionnaire (GHQ)		
Jamilian et al., 2018 [9]	NR	↓ ^1^
Ostadmohammadi et al., 2019 [6]	NR	↓ ^1^
12-item Short Form Health Survey (SF-12)		
Hariri et al., 2024 [21]	Physical score	Physical score
	NR	↔ ^1^
	Mental score	Mental score
	NR	↔ ^1^
Perceived Stress Scale (PSS-10)		
Hariri et al., 2024 [21]	SG: ↔ PG: ↔	↔ ^1^

SG—supplemented group; PG—placebo group; NR—not reported; ↔—without changes before and after intervention; ↓—decreased after intervention; ↑—increased after intervention; ↔ ^1^—no differences between SG and PG; ↓ ^1^—difference in outcome measures between SG and PG (where the results in SG were lower); ↑ ^1^—difference in outcome measures between SG and PG (where the results in SG were higher).

## Data Availability

The original contributions presented in the study are included in the article/Supplementary Materials, further inquiries can be directed to the corresponding author.

## Supplementary Materials

The following supporting information can be downloaded at: https://www.mdpi.com/article/10.3390/nu18020307/s1, Table S1: Evaluation of the studies’ quality. File S1: PRISMA Checklist. Ref. [44] is cited in Supplementary Materials file.

## Abbreviations

The following abbreviations are used in this manuscript:

PCOS	Polycystic ovary syndrome
RCT	Randomized controlled trial
PICO	Population, Intervention, Control, and Outcome
BMI	Body mass index
DASS-21	Depression, Anxiety, and Stress Scale-21 items
PCOSQ-26	Polycystic Ovary Syndrome Health-related Quality of Life Questionnaire
SF-12	12-item Short-form Health Survey
PSS-10	Perceived Stress Scale
BDI	Beck Depression Inventory
GHQ-28	General Health Questionnaire-28
PSQI	Pittsburgh Sleep Quality Index
SG	Supplemented group
PG	Placebo group

## References

[1] Salari N., Nankali A., Ghanbari A., Jafarpour S., Ghasemi H., Dokaneheifard S., Mohammadi M. (2024). Global prevalence of polycystic ovary syndrome in women worldwide: A comprehensive systematic review and meta-analysis. Arch. Gynecol. Obs..

[2] Yu Z., Qin E., Cheng S., Yang H., Liu R., Xu T., Liu Y., Yuan J., Yu S., Yang J. (2022). Gut microbiome in PCOS associates to serum metabolomics: A cross-sectional study. Sci. Rep..

[3] Kitzinger C., Willmott J. (2002). ‘The thief of womanhood’: Women’s experience of polycystic ovarian syndrome. Soc. Sci. Med..

[4] Gnawali A., Patel V., Cuello-Ramírez A., Al Kaabi A.S., Noor A., Rashid M.Y., Henin S., Mostafa J.A. (2021). Why are Women with Polycystic Ovary Syndrome at Increased Risk of Depression? Exploring the Etiological Maze. Cureus.

[5] Li H., Liu M., Zhang C. (2022). Women with polycystic ovary syndrome (PCOS) have reduced melatonin concentrations in their follicles and have mild sleep disturbances. BMC Women’s Health.

[6] Ostadmohammadi V., Jamilian M., Bahmani F., Asemi Z. (2019). Vitamin D and probiotic co-supplementation affects mental health, hormonal, inflammatory and oxidative stress parameters in women with polycystic ovary syndrome. J. Ovarian Res..

[7] Samimi M., Dadkhah A., Haddad Kashani H., Tajabadi-Ebrahimi M., Seyed Hosseini E., Asemi Z. (2019). The Effects of Synbiotic Supplementation on Metabolic Status in Women with Polycystic Ovary Syndrome: A Randomized Double-Blind Clinical Trial. Probiotics Antimicrob. Prot..

[8] Tabrizi R., Ostadmohammadi V., Akbari M., Lankarani K.B., Vakili S., Peymani P., Karamali M., Kolahdooz F., Asemi Z. (2022). The Effects of Probiotic Supplementation on Clinical Symptom, Weight Loss, Glycemic Control, Lipid and Hormonal Profiles, Biomarkers of Inflammation, and Oxidative Stress in Women with Polycystic Ovary Syndrome: A Systematic Review and Meta-analysis of Randomized Controlled Trials. Probiotics Antimicrob. Prot..

[9] Jamilian M., Mansury S., Bahmani F., Heidar Z., Amirani E., Asemi Z. (2018). The effects of probiotic and selenium co-supplementation on parameters of mental health, hormonal profiles, and biomarkers of inflammation and oxidative stress in women with polycystic ovary syndrome. J. Ovarian Res..

[10] Karamali M., Eghbalpour S., Rajabi S., Jamilian M., Bahmani F., Tajabadi-Ebrahimi M., Keneshlou F., Mirhashemi S.M., Chamani M., Hashem Gelougerdi S. (2018). Effects of Probiotic Supplementation on Hormonal Profiles, Biomarkers of Inflammation and Oxidative Stress in Women with Polycystic Ovary Syndrome: A Randomized, Double-Blind, Placebo-Controlled Trial. Arch. Iran. Med..

[11] Liao D., Zhong C., Li C., Mo L., Liu Y. (2018). Meta-analysis of the effects of probiotic supplementation on glycemia, lipidic profiles, weight loss and C-reactive protein in women with polycystic ovarian syndrome. Minerva Med..

[12] Lv T., Ye M., Luo F., Hu B., Wang A., Chen J., Yan J., He Z., Chen F., Qian C. (2021). Probiotics treatment improves cognitive impairment in patients and animals: A systematic review and meta-analysis. Neurosci. Biobehav. Rev..

[13] Mo R., Jiang M., Xu H., Jia R. (2024). Effect of probiotics on cognitive function in adults with mild cognitive impairment or Alzheimer’s disease: A meta-analysis of randomized controlled trials. Med. Clínica.

[14] Dokras A. (2025). Polycystic ovary syndrome in 2025—Insights and innovations. Fertil. Steril..

[15] Naz M.S.G., Rahnemaei F.A., Tehrani F.R., Sayehmiri F., Ghasemi V., Banaei M., Ozgoli G. (2023). Possible cognition changes in women with polycystic ovary syndrome: A narrative review. Obs. Gynecol. Sci..

[16] Bernstein M.E., Dokras A., Flaherty C. (2025). Neuropsychological profile of polycystic ovary syndrome: Past, present, and future. Fertil. Steril..

[17] Zandifar A., Badrfam R., Mohammaditabar M., Kargar B., Goodarzi S., Hajialigol A., Ketabforoush S., Heidari A., Fathi H., Shafiee A. (2025). The Effect of Prebiotics and Probiotics on Levels of Depression, Anxiety, and Cognitive Function: A Meta-Analysis of Randomized Clinical Trials. Brain Behav..

[18] Liu R., Zhang C., Shi Y., Zhang F., Li L., Wang X., Ling Y., Fu H., Dong W., Shen J. (2017). Dysbiosis of Gut Microbiota Associated with Clinical Parameters in Polycystic Ovary Syndrome. Front. Microbiol..

[19] Page M.J., McKenzie J.E., Bossuyt P.M., Boutron I., Hoffmann T.C., Mulrow C.D., Shamseer L., Tetzlaff J.M., Akl E.A., Brennan S.E. (2021). The PRISMA 2020 statement: An updated guideline for reporting systematic reviews. BMJ.

[20] Sterne J.A.C., Savović J., Page M.J., Elbers R.G., Blencowe N.S., Boutron I., Cates C.J., Cheng H.-Y., Corbett M.S., Eldridge S.M. (2019). RoB 2: A revised tool for assessing risk of bias in randomised trials. BMJ.

[21] Hariri Z., Yari Z., Hoseini S., Abhari K., Sohrab G. (2024). Synbiotic as an ameliorating factor in the health-related quality of life in women with polycystic ovary syndrome. A randomized, triple-blind, placebo-controlled trial. BMC Women’s Health.

[22] Askarpour M., Hejazi N., Jahromi B.N., Eskandari M.H., Famouri M., Bedeltavana A. (2025). Effects of a Novel Fortified Dairy Product on the Psychological Status and Sleep Quality of Patients with Polycystic Ovary Syndrome: A Double-Blind Randomized Controlled Trial. Prev. Nutr. Food Sci..

[23] Kaur I., Suri V., Sachdeva N., Rana S.V., Medhi B., Sahni N., Ahire J., Singh A. (2022). Efficacy of multi-strain probiotic along with dietary and lifestyle modifications on polycystic ovary syndrome: A randomised, double-blind placebo-controlled study. Eur. J. Nutr..

[24] Zheng P., Zeng B., Zhou C., Liu M., Fang Z., Xu X., Zeng L., Chen J., Fan S., Du X. (2016). Gut microbiome remodeling induces depressive-like behaviors through a pathway mediated by the host’s metabolism. Mol. Psychiatry.

[25] Konturek S.J., Konturek J.W., Pawlik T., Brzozowski T. (2004). Brain-gut axis and its role in the control of food intake. J. Physiol. Pharmacol..

[26] Schneider E., Doll J.P.K., Schweinfurth N., Kettelhack C., Schaub A.-C., Yamanbaeva G., Varghese N., Mählmann L., Brand S., Eckert A. (2023). Effect of short-term, high-dose probiotic supplementation on cognition, related brain functions and BDNF in patients with depression: A secondary analysis of a randomized controlled trial. J. Psychiatry Neurosci..

[27] Cooney L.G., Lee I., Sammel M.D., Dokras A. (2017). High prevalence of moderate and severe depressive and anxiety symptoms in polycystic ovary syndrome: A systematic review and meta-analysis. Hum. Reprod..

[28] Dubé-Zinatelli E., Anderson F., Ismail N. (2025). The overlooked mental health burden of polycystic ovary syndrome: Neurobiological insights into PCOS-related depression. Front. Neuroendocrinol..

[29] Fernandez R., Moore V., Van Ryswyk E., Varcoe T., Rodgers R., March W., Moran L., Avery J., McEvoy D., Davies M. (2018). Sleep disturbances in women with polycystic ovary syndrome: Prevalence, pathophysiology, impact and management strategies. Nat. Sci. Sleep.

[30] Jafar N.K.A., Fan M., Moran L.J., Mansfield D.R., Bennett C.J. (2025). Sex Hormones, Sex Hormone-Binding Globulin and Sleep Problems in Females With Polycystic Ovary Syndrome: A Systematic Review and Meta-Analysis. Clin. Endocrinol..

[31] Dziedzic A., Maciak K., Bliźniewska-Kowalska K., Gałecka M., Kobierecka W., Saluk J. (2024). The Power of Psychobiotics in Depression: A Modern Approach through the Microbiota–Gut–Brain Axis: A Literature Review. Nutrients.

[32] Cederberg K.L.J., Hanif U., Peris Sempere V., Hédou J., Leary E.B., Schneider L.D., Lin L., Zhang J., Morse A.M., Blackman A. (2022). Proteomic Biomarkers of the Apnea Hypopnea Index and Obstructive Sleep Apnea: Insights into the Pathophysiology of Presence, Severity, and Treatment Response. Int. J. Mol. Sci..

[33] Beroukhim G., Esencan E., Seifer D.B. (2022). Impact of sleep patterns upon female neuroendocrinology and reproductive outcomes: A comprehensive review. Reprod. Biol. Endocrinol..

[34] Bennett C.J., Mansfield D.R., Mo L., Joham A.E., Cain S.W., Blumfield M.L., Hodge A.M., Moran L.J. (2022). Sleep disturbances may influence lifestyle behaviours in women with self-reported polycystic ovary syndrome. Br. J. Nutr..

[35] Ho Y.-T., Tsai Y.-C., Kuo T.B.J., Yang C.C.H. (2021). Effects of Lactobacillus plantarum PS128 on Depressive Symptoms and Sleep Quality in Self-Reported Insomniacs: A Randomized, Double-Blind, Placebo-Controlled Pilot Trial. Nutrients.

[36] Sobol-Kwapinska M., Jankowski T. (2016). Positive Time: Balanced Time Perspective and Positive Orientation. J. Happiness Stud..

[37] Brady C., Mousa S.S., Mousa S.A. (2009). Polycystic ovary syndrome and its impact on women’s quality of life: More than just an endocrine disorder. Drug Healthc. Patient Saf..

[38] Hollinrake E., Abreu A., Maifeld M., Van Voorhis B.J., Dokras A. (2007). Increased risk of depressive disorders in women with polycystic ovary syndrome. Fertil. Steril..

[39] Sanchez M., Darimont C., Panahi S., Drapeau V., Marette A., Taylor V., Doré J., Tremblay A. (2017). Effects of a Diet-Based Weight-Reducing Program with Probiotic Supplementation on Satiety Efficiency, Eating Behaviour Traits, and Psychosocial Behaviours in Obese Individuals. Nutrients.

[40] Raygan F., Ostadmohammadi V., Bahmani F., Asemi Z. (2018). The effects of vitamin D and probiotic co-supplementation on mental health parameters and metabolic status in type 2 diabetic patients with coronary heart disease: A randomized, double-blind, placebo-controlled trial. Prog. Neuro-Psychopharmacol. Biol. Psychiatry.

[41] Ahmad S.R., AlShahrani A.M., Kumari A. (2025). Effects of Probiotic Supplementation on Depressive Symptoms, Sleep Quality, and Modulation of Gut Microbiota and Inflammatory Biomarkers: A Randomized Controlled Trial. Brain Sci..

[42] Yang Y., Yang L., Wan M., Pan D., Sun G., Yang C. (2024). Assessment of optimal combinations of therapeutic probiotics for depression, anxiety, and stress. Psychol. Med..

[43] Liu R.T., Walsh R.F.L., Sheehan A.E. (2019). Prebiotics and probiotics for depression and anxiety: A systematic review and meta-analysis of controlled clinical trials. Neurosci. Biobehav. Rev..

[44] Page M.J., McKenzie J.E., Bossuyt P.M., Boutron I., Hoffmann T.C., Mulrow C.D., Shamseer L., Tetzlaff J., Akl E., Brennan S.E. (2020). The PRISMA 2020 statement: An updated guideline for reporting systematic reviews. MetaArXiv.

