# Supplementary material for: Effects of Probiotic Supplementation on Mental Health and the Risk of Depression in Women with Polycystic Ovary Syndrome: A Systematic Review of Randomized Controlled Trials
Source: Nutrients. 2026 Jan 19;18(2):307. doi: 10.3390/nu18020307 (PMC12844778; doi:10.3390/nu18020307)
Supplement: Supplementary file 1 [file nutrients-18-00307-s001.zip › Table S1.pdf]

Supplementary Table S1. Evaluation of the studies quality.

**Author, Year**	**Randomization process**	**Deviations from intended interventions**	**Missing outcome data**	**Measurement of the outcome**	**Selection of the reported result**	**Overall bias**
Askarpour et al. 2025	Some concerns	Low	Some concerns	Low	Some concerns	Some concerns
Hariri et al. 2024	Some concerns	Low	Some concerns	High	High	High
Jamilian et al. 2018	High	Low	Low	High	Low	High
Ostadmohammadi et al. 2019	High	Low	Low	Some concerns	Low	Some concerns
Kaur et al. 2022	High	Low	Some concerns	Some concerns	Low	Some concerns
